# Global pharmacovigilance analysis of dermatologic toxicity in EGFR-TKI–treated NSCLC: a cross-database real-world study

**DOI:** 10.3389/fphar.2026.1789598

**Published:** 2026-08-12

**Authors:** Natalia Sauer, Piotr Giedziun, Jacek Calik, Anna Wiela-Hojeńska

**Affiliations:** 1 Department of Clinical Pharmacology, Faculty of Pharmacy, Wroclaw Medical University, Wroclaw, Poland; 2 Department of Artificial Intelligence, Wroclaw University of Science and Technology, Wroclaw, Poland; 3 Old Town Clinic, Wrocław, Poland

**Keywords:** ADR (adverse drug reaction), EGFR- TKI, Eudra Vigilance, pharmacovgilance, skin toxicity

## Abstract

**Introduction:**

Epidermal growth factor receptor tyrosine kinase inhibitors (EGFR-TKIs) have transformed the treatment of EGFR-mutant non–small-cell lung cancer (NSCLC), significantly improving survival outcomes and quality of life compared with conventional chemotherapy. However, inhibition of EGFR signaling, a key regulator of epidermal homeostasis, frequently results in dermatologic adverse drug reactions (ADRs), which may compromise treatment adherence and negatively affect patient well-being. This study aimed to characterize the real-world reporting patterns of cutaneous ADRs associated with five EGFR-TKIs using large-scale pharmacovigilance databases.

**Methods:**

A retrospective pharmacovigilance analysis was performed using the World Health Organization VigiAccess™ database. Reports associated with gefitinib, erlotinib, afatinib, osimertinib, and lazertinib were extracted and evaluated. Dermatologic ADRs included disorders of the skin, subcutaneous tissue, hair, nails, and mucosa. Reporting frequencies and disproportionality analyses were conducted to compare dermatologic toxicity profiles across agents. To validate the findings and assess reproducibility, an independent analysis was additionally performed using the European Medicines Agency (EMA) EudraVigilance database.

**Results:**

A total of 96,386 ADR reports were identified, of which 25,383 (29.7%) involved dermatologic manifestations. Rash and pruritus were the most frequently reported cutaneous ADRs across all EGFR-TKIs, although their proportional distribution differed between agents. Erlotinib and afatinib demonstrated the highest proportions of dermatologic reports (30.8% and 43.0%, respectively), whereas osimertinib exhibited a lower relative frequency (11.6%) but a greater proportion of nail and hair disorders within the dermatologic category. Lazertinib displayed a distinct reporting pattern characterized by frequent rash and pruritus signals, while maintaining an overall dermatologic profile consistent with the class effect of EGFR inhibition. Disproportionality analyses revealed statistically significant differences in reporting patterns among individual EGFR-TKIs. Comparable trends were observed in the EudraVigilance database, supporting the reproducibility and external validity of the identified safety signals.

**Discussion:**

Large-scale pharmacovigilance data demonstrate that although dermatologic toxicity represents a class effect of EGFR inhibition, substantial differences exist in the spectrum and relative frequency of cutaneous ADRs among individual EGFR-TKIs. Cross-validation using independent pharmacovigilance systems strengthens the reliability of these findings and underscores the importance of agent-specific toxicity surveillance. These results support proactive multidisciplinary management strategies aimed at minimizing treatment interruptions, optimizing therapeutic adherence, and improving patient-centered care in individuals with EGFR-mutant NSCLC.

## Introduction

Non–small-cell lung cancer (NSCLC) remains the leading cause of cancer-related mortality worldwide, and a substantial subset of patients presents with activating mutations in the epidermal growth factor receptor (EGFR) gene (Sung et al., 2021; Zhang et al., 2016). These mutations drive tumorigenesis and confer sensitivity to a class of targeted therapies known as EGFR tyrosine kinase inhibitors (EGFR-TKIs) (Zhou et al., 2025). As systemic treatment in advanced disease settings, EGFR-TKIs have substantially transformed the therapeutic landscape for EGFR-mutant NSCLC, improving progression-free survival and quality of life compared with conventional chemotherapy (Mok et al., 2009; Sequist et al., 2013).

However, the molecular mechanism underlying their therapeutic effect—selective inhibition of EGFR signaling—also disrupts physiological processes in normal tissues, particularly the skin (Laface et al., 2023; Cross et al., 2014). EGFR is highly expressed in keratinocytes and sebaceous glands and plays a central role in skin barrier integrity, wound healing, and follicular homeostasis (Holcmann and Sibilia, 2015; Al-Dhubaibi et al., 2025). Consequently, pharmacologic blockade commonly leads to characteristic cutaneous adverse events such as papulopustular rash, xerosis, pruritus, paronychia, and alopecia (Kozuki, 2016; Li et al., 2022). These toxicities are not only frequent, but may also be dose-limiting, psychologically distressing, and detrimental to treatment adherence and patient-reported quality of life (Du et al., 2022).

The severity and spectrum of these reactions vary according to the generation and selectivity of the EGFR-TKI used (Karachaliou et al., 2019; Wang and Li, 2019; Ma et al., 2024). First-generation inhibitors (gefitinib, erlotinib) and second-generation agents (afatinib) inhibit both mutant and wild-type EGFR more broadly, often resulting in a higher burden of dermatologic toxicity (Shaban et al., 2024). In contrast, third-generation TKIs such as osimertinib and lazertinib were designed to preferentially target mutant EGFR and resistance-associated alterations such as T790M while sparing wild-type EGFR to a greater extent, thereby offering a more favorable toxicity profile (Li et al., 2023). Nevertheless, skin reactions remain clinically relevant even with these newer agents.

Although treatment selection in EGFR-mutant NSCLC is primarily driven by molecular subtype, line of therapy, and expected efficacy, toxicity profiles remain clinically important because they influence treatment persistence, dose intensity, supportive care burden, and the feasibility of subsequent therapeutic sequencing (National Comprehensive Cancer Network, 2026; Puri et al., 2026; Li et al., 2024; Ding et al., 2017; Lee et al., 2024; Yu et al., 2020; Hsu et al., 2022; Seki et al., 2025). Current guideline frameworks identify osimertinib as the preferred first-line EGFR TKI for common sensitizing EGFR mutations, whereas earlier-generation agents are generally reserved for selected clinical circumstances; however, this hierarchy does not eliminate the practical importance of adverse-event profiles when choosing among active options, particularly in older or frail patients, in those with uncommon EGFR mutations, and in patients with pre-existing organ vulnerabilities (National Comprehensive Cancer Network, 2026; Puri et al., 2026). In settings where efficacy is broadly comparable within a treatment line, toxicity may become a major differentiating factor, as afatinib and erlotinib are consistently associated with higher rates of severe treatment-related adverse events than gefitinib or osimertinib (Li et al., 2024; Ding et al., 2017). This is especially relevant for dermatologic toxicity, which is among the most frequent class-specific adverse effects of EGFR inhibition and a documented cause of dose reduction, treatment interruption, and quality-of-life impairment (Lee et al., 2024; Yu et al., 2020; Hsu et al., 2022). Moreover, the first-line choice of EGFR TKI has implications for downstream management at progression, including T790M-directed sequencing and the use of combination regimens with distinct prophylactic and monitoring requirements (National Comprehensive Cancer Network, 2026; Seki et al., 2025).

Given the early onset and cumulative impact of dermatologic toxicities, timely recognition and continuous monitoring are essential components of patient management (Melosky et al., 2015; Kafatos et al., 2020). Pharmacovigilance data play a central role in identifying drug-specific safety signals and refining post-marketing safety profiles (Yin et al., 2022). Importantly, adverse-event patterns observed in large-scale pharmacovigilance datasets may reveal population-level trends not fully captured in clinical trials, thereby informing real-world toxicity surveillance and supportive care strategies. In this context, oncology pharmacists contribute not only to adverse-event detection and patient education, but also to adherence support and the quality of toxicity reporting, alongside oncologists and dermatologists within a multidisciplinary care model (Al-Quteimat and Amer, 2020; Alkofide et al., 2024; Baldo et al., 2018; Boşnak et al., 2018).

In this study, we performed a comparative analysis of dermatologic adverse drug reactions associated with five EGFR-TKIs—gefitinib, erlotinib, afatinib, osimertinib, and lazertinib—using real-world pharmacovigilance data. We aimed to characterize drug-specific cutaneous safety profiles, examine how these patterns relate to EGFR-TKI selectivity, and assess the extent to which findings are consistent across two major pharmacovigilance systems. By doing so, we sought to generate clinically relevant real-world evidence that may support individualized toxicity monitoring and multidisciplinary supportive care in patients with EGFR-mutant NSCLC.

## Materials and methods

### Data source and study design

This retrospective pharmacovigilance study was conducted using data obtained from VigiAccess™, a public interface for the World Health Organization (WHO) global database of individual case safety reports (ICSRs) known as VigiBase®. VigiBase is maintained by the Uppsala Monitoring Centre (UMC) and collects anonymized reports of suspected adverse drug reactions (ADRs) submitted by national pharmacovigilance authorities in over 130 countries. VigiAccess provides aggregated summaries of reported ADRs by drug name and classification, utilizing the standardized MedDRA (Medical Dictionary for Regulatory Activities) terminology. To strengthen the validity and generalizability of our findings, we conducted a comparative analysis using data from both WHO’s VigiAccess and the EMA’s EudraVigilance pharmacovigilance databases. While the primary analysis was based on VigiAccess due to its broader international scope and higher volume of reports, EudraVigilance offered an independent and regionally focused dataset for cross-validation.

### Selection of drugs and data extraction

The analysis focused on five epidermal growth factor receptor tyrosine kinase inhibitors (EGFR-TKIs) currently used in the treatment of advanced EGFR-mutant non-small cell lung cancer (NSCLC): gefitinib, erlotinib, afatinib, osimertinib, and lazertinib (Table 1). These agents were selected to represent first-, second-, and third-generation EGFR-targeted therapies. In May 2025, VigiAccess was queried using the international non-proprietary name (INN) of each drug.

**TABLE 1:** Overview of five epidermal growth factor receptor tyrosine kinase inhibitors (EGFR-TKIs) used in the treatment of advanced EGFR-mutant non-small cell lung cancer (NSCLC).

Active ingredient	Generation	Brand names	Chemical formula	Drug targets	Main conditions	The earliest year on the market
Gefitinib	1st generation	Iressa, Gefinib, Gefonib, Zegerin, Geftib	C22H24ClFN4O3	EGFR (wild-type and mutant)	Non-small cell lung cancer (NSCLC)	2003
Erlotinib	1st generation	Tarceva, Erlonat, Erleva, Erlibelle, Erolin	C22H23N3O4	EGFR (wild-type and mutant)	NSCLC, pancreatic cancer	2004
Afatinib	2nd generation	Gilotrif, Giotrif, Afanix	C24H25ClFN5O3	EGFR (irreversible inhibitor)	NSCLC (EGFR-mutant)	2013
Osimertinib	3rd generation	Tagrisso, Tagrix, Osicent, Azletinib	C28H33N7O2F2	EGFR T790M mutation	EGFR T790M-positive NSCLC	2015
Lazertinib	3rd generation	Leclaza	C30H29FN6O2	EGFR T790M mutation	EGFR T790M-positive NSCLC	2021

For each EGFR-TKI, the total number of ICSRs was recorded, along with the number of reports classified under categories relevant to cutaneous and mucocutaneous adverse events. Specifically, we included ADRs listed under the MedDRA system organ classes (SOCs) corresponding to skin and subcutaneous tissue disorders, nail disorders, hair disorders, and mucosal disorders. Preferred Terms (PTs) included, but were not limited to, acneiform rash, xerosis, pruritus, erythema, paronychia, nail dystrophy, alopecia, trichomegaly, stomatitis, oral ulcers, and mucositis. Data were extracted as aggregate values as provided by the VigiAccess interface; due to the anonymized and cumulative nature of the data, individual case details and duplicate reporting could not be assessed or adjusted for.

### Data analysis

Descriptive statistics were used to evaluate the frequency and distribution of cutaneous ADRs associated with each EGFR-TKI. The proportion of cutaneous and mucocutaneous events relative to the total number of reported ADRs for each drug was calculated. Additionally, the distribution of specific adverse event types was compared across agents to explore potential differences in safety profiles.

To quantify the disproportionality of reporting for specific dermatologic events, we calculated the Reporting Odds Ratio (ROR) for each Preferred Term. ROR was defined as the odds of a specific ADR being reported for a given drug relative to all other cutaneous ADRs for that drug, divided by the odds of any cutaneous ADR being reported among all other (non-cutaneous) ADRs for the same drug. The formula used was:

$$ROR=\frac{a/b}{c/d}$$

Where:

- **a** = number of reports of the specific dermatologic ADR (Preferred Term) for the drug of interest
- **b** = number of reports of the same ADR for all other drugs
- **c** = number of reports of other ADRs (except the specific Preferred Term) for the drug of interest
- **d** = number of reports of all other ADRs for all other drugs

This approach compares the odds of reporting a specific dermatologic ADR for a given drug to the odds of reporting the same ADR for all other drugs in the database. An ROR > 1 suggests that the ADR is reported more frequently than expected and may indicate a signal that warrants further investigation.

In parallel, Proportional Reporting Ratio (PRR) was calculated to measure the relative proportion of a given ADR among all reports for one drug compared to that proportion among all other drugs. The formula was:

$$PRR=\frac{a/\left(a+b\right)}{c/\left(c+d\right)}$$

A PRR value greater than 1 suggests a higher-than-expected frequency of the given ADR for the drug of interest relative to the background frequency. Both ROR and PRR were reported along with their 95% confidence intervals (CI), which were calculated using logarithmic transformation and standard error estimation methods.

Given the limitations of spontaneous reporting systems, including underreporting, differences in regional reporting practices, lack of exposure data, and the inability to confirm causality - these findings should be interpreted as exploratory signals rather than definitive evidence of risk.

## Results

As of May 11, 2025, the WHO VigiAccess database contained a total of 96,386 adverse drug reaction (ADR) reports associated with five EGFR tyrosine kinase inhibitors (TKIs): Gefitinib (10,303), Erlotinib (42,765), Afatinib (12,498), Osimertinib (29,170), and Lazertinib (1,650) (Table 1). The earliest ADR reports were recorded for Erlotinib in 2003, while Lazertinib entries first appeared in 2021, reflecting its relatively recent clinical introduction.

**FIGURE 1:**
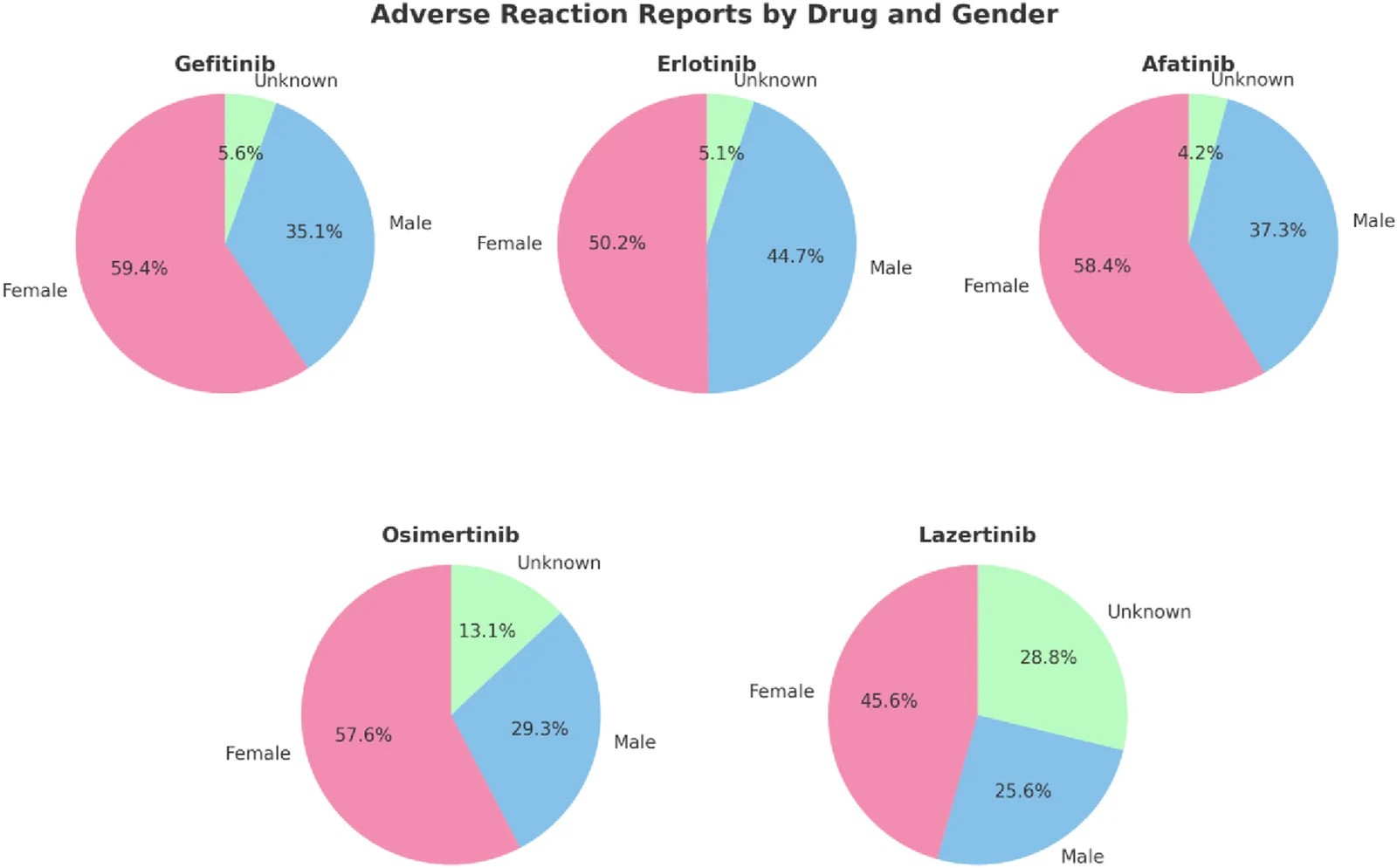
Distribution of adverse drug reaction (ADR) reports by gender for five EGFR-targeted tyrosine kinase inhibitors.

The sex distribution, after excluding 5,079 reports with unknown gender, includes 52,447 ADRs in females (59.07%) and 36,360 ADRs in males (40.93%), yielding a female-to-male ratio of 1.44:1 (Figure 1). Demographic and geographic distribution of adverse drug reaction (ADR) reports for five EGFR-targeted tyrosine kinase inhibitors are shown in Table 2. A chi-square test confirmed that this distribution significantly deviates from the expected 1:1 ratio (χ^2^ = 2,914.1, p < 0.001), indicating a notable predominance of ADRs in female patients. This may be related to known epidemiological patterns, where EGFR mutations - and thus TKI indications are more common among women, particularly non-smokers.

**TABLE 2:** Demographic and geographic distribution of adverse drug reaction (ADR) reports for five EGFR-targeted tyrosine kinase inhibitors.

Characteristic	Gefitynib	Erlotinib	Afatinib	Osimertinib	Lazertinib
Number of ADR reports	10,303 (100.0%)	42,765 (100.0%)	12,498 (100.0%)	29,170 (100.0%)	1,650 (100.0%)
Female	6,116 (59.4%)	21,459 (50.2%)	7,304 (58.4%)	16,815 (57.6%)	753 (45.6%)
Male	3,614 (35.1%)	19,123 (44.7%)	4,668 (37.3%)	8,533 (29.3%)	422 (25.6%)
Unknown	573 (5.6%)	2,183 (5.1%)	526 (4.2%)	3,822 (13.1%)	475 (28.8%)
0–27 days	3 (0.0%)	8 (0.0%)	4 (0.0%)	19 (0.1%)	–
28 days to 23 months	2 (0.0%)	11 (0.0%)	3 (0.0%)	1 (0.0%)	–
2–11 years	21 (0.2%)	39 (0.1%)	12 (0.1%)	6 (0.0%)	–
12–17 years	11 (0.1%)	37 (0.1%)	2 (0.0%)	2 (0.0%)	–
< 18 years	–	–	–	–	–
18–44 years	446 (4.3%)	856 (2.0%)	489 (3.9%)	532 (1.8%)	35 (2.1%)
45–64 years	2,927 (28.4%)	7,620 (17.8%)	4,148 (33.2%)	5,413 (18.6%)	487 (29.5%)
65–74 years	2,606 (25.3%)	6,857 (16.0%)	3,317 (26.5%)	5,665 (19.4%)	483 (29.3%)
≥ 75 years	2093 (20.3%)	5,288 (12.4%)	2,367 (18.9%)	5,698 (19.5%)	474 (28.7%)
Unknown age	2,194 (21.3%)	22,049 (51.6%)	2,156 (17.3%)	11,834 (40.6%)	171 (10.4%)
Africa	12 (0.1%)	77 (0.2%)	14 (0.1%)	72 (0.2%)	0 (0.0%)
Americas	2,721 (26.4%)	31,513 (73.7%)	2,418 (19.3%)	13,090 (44.9%)	119 (7.2%)
Asia	5,775 (56.1%)	4,640 (10.8%)	6,825 (54.6%)	11,114 (38.1%)	1,517 (91.9%)
Europe	1,639 (15.9%)	6,330 (14.8%)	3,210 (25.7%)	4,486 (15.4%)	5 (0.3%)
Oceania	156 (1.5%)	205 (0.5%)	31 (0.2%)	408 (1.4%)	9 (0.5%)
2025	61 (0.6%)	111 (0.3%)	187 (1.5%)	2,327 (8.0%)	748 (45.3%)
2024	540 (5.2%)	640 (1.5%)	630 (5.0%)	7,818 (26.8%)	687 (41.6%)
2023	463 (4.5%)	541 (1.3%)	331 (2.6%)	4,516 (15.5%)	206 (12.5%)
2022	309 (3.0%)	373 (0.9%)	291 (2.3%)	3,535 (12.1%)	7 (0.4%)
2021	603 (5.9%)	513 (1.2%)	1,053 (8.4%)	3,649 (12.5%)	2 (0.1%)
2020	895 (8.7%)	580 (1.4%)	2,118 (16.9%)	2,687 (9.2%)	0 (0.0%)
2019	651 (6.3%)	1,571 (3.7%)	1,628 (13.0%)	2,156 (7.4%)	0 (0.0%)
2018	583 (5.7%)	2,599 (6.1%)	1904 (15.2%)	1,284 (4.4%)	0 (0.0%)
2017	813 (7.9%)	8,334 (19.5%)	1,495 (12.0%)	940 (3.2%)	0 (0.0%)
2016	633 (6.1%)	1847 (4.3%)	1,290 (10.3%)	250 (0.9%)	0 (0.0%)
2015	430 (4.2%)	4,673 (10.9%)	924 (7.4%)	5 (0.0%)	0 (0.0%)
2014	552 (5.4%)	3,715 (8.7%)	290 (2.3%)	3 (0.0%)	0 (0.0%)
2013	491 (4.8%)	7,978 (18.7%)	188 (1.5%)	–	–
Before 2013	5 (0.0%)	–	–	–	–

Age group analysis, excluding 31,738 reports with unknown age, reveals the highest frequency of ADRs among patients aged 45–64 and 65–74 years, consistent with the typical age distribution of lung cancer. Pediatric reports (0–17 years) accounted for less than 0.5% of all cases.

Geographically, the majority of ADRs originated from the Americas (approx. 47.5%), Asia (32.6%), and Europe (18.1%), reflecting both regional drug usage patterns and differences in pharmacovigilance infrastructure. Of note, Lazertinib reports were overwhelmingly from Asia (92%), suggesting either restricted global availability or regional concentration in clinical practice. ADR reporting trends show a marked increase for Osimertinib and Lazertinib in recent years, likely reflecting their rising usage as standard-of-care agents in EGFR-mutated non-small cell lung cancer.

### Distribution of 20 SOCs

A detailed analysis of the distribution of 20 System Organ Classes (SOCs) was conducted for five EGFR-targeted tyrosine kinase inhibitors (Supplementary Table S1). Across all agents—gefitinib, erlotinib, afatinib, osimertinib, and lazertinib—the highest proportions of adverse drug reaction (ADR) reports were consistently observed in gastrointestinal disorders, general disorders and administration site conditions, and skin and subcutaneous tissue disorders.

Afatinib showed the highest proportional representation of gastrointestinal disorder reports (25%), whereas lazertinib demonstrated a distinct reporting pattern characterized by a higher proportion of nervous system disorder reports (19%) compared with the other agents (≤5%), with statistically significant inter-drug variation (χ^2^ = 2,736.1, p < 0.001).

Skin and subcutaneous tissue disorder reports accounted for 19% of afatinib ICSRs, 17% of both gefitinib and lazertinib ICSRs, and 7% of osimertinib ICSRs, with a statistically significant difference across agents (χ^2^ (4) = 3,049.4, p < 0.001, Cramér’s V = 0.09). Respiratory disorder reports were proportionally most frequent with osimertinib (10% vs. 6%–7% for the remaining agents), also demonstrating significant inter-drug variation (χ^2^ (4) = 715.3, p < 0.001).

Overall, the distribution of adverse-event SOC categories differed significantly across the five EGFR-TKIs (χ^2^ (76) = 17,672.60, p < 0.001, Cramér’s V = 0.16), supporting the relevance of agent-specific pharmacovigilance-based safety monitoring strategies.

### Most common ADRs

Supplementary Table S2 summarizes the 20 most frequently reported adverse drug reactions (ADRs) for five EGFR tyrosine kinase inhibitors (TKIs): gefitinib, erlotinib, afatinib, osimertinib, and lazertinib, based on VigiAccess data. Across all agents, the most commonly reported ADRs included diarrhoea, rash, pruritus, nausea, vomiting, decreased appetite, dyspnoea, asthenia, fatigue, pneumonia, and dry skin. Several of these reactions—particularly dermatologic and gastrointestinal events—may affect quality of life and treatment adherence and therefore remain clinically relevant during EGFR-TKI therapy.

Afatinib demonstrated the highest proportional representation of gastrointestinal and mucocutaneous ADR reports, including diarrhoea, rash, stomatitis, and paronychia, consistent with its broader inhibition of the ErbB receptor family. Erlotinib and osimertinib showed relatively high proportions of reports with fatal outcomes; however, these entries represent report-level seriousness outcomes recorded in spontaneous reporting systems and should not be interpreted as confirmed treatment-related adverse reactions. Instead, they likely reflect differences in treatment setting, disease stage, or reporting structure rather than drug-attributable toxicity.

Interstitial lung disease appeared among the most frequently reported ADRs for osimertinib and remains a clinically important safety signal requiring active monitoring in routine practice. Lazertinib demonstrated a distinct reporting pattern characterized by relatively frequent reports of rash and paraesthesia, suggesting differences in neurologic adverse-event reporting compared with earlier-generation EGFR-TKIs.

Eight ADRs—diarrhoea, rash, pruritus, nausea, vomiting, decreased appetite, asthenia, and fatigue—were reported among the top 20 events for at least four of the five agents, supporting a consistent class-effect pattern of frequently reported EGFR-TKI–associated adverse reactions.

### Serious adverse events

**FIGURE 2:**
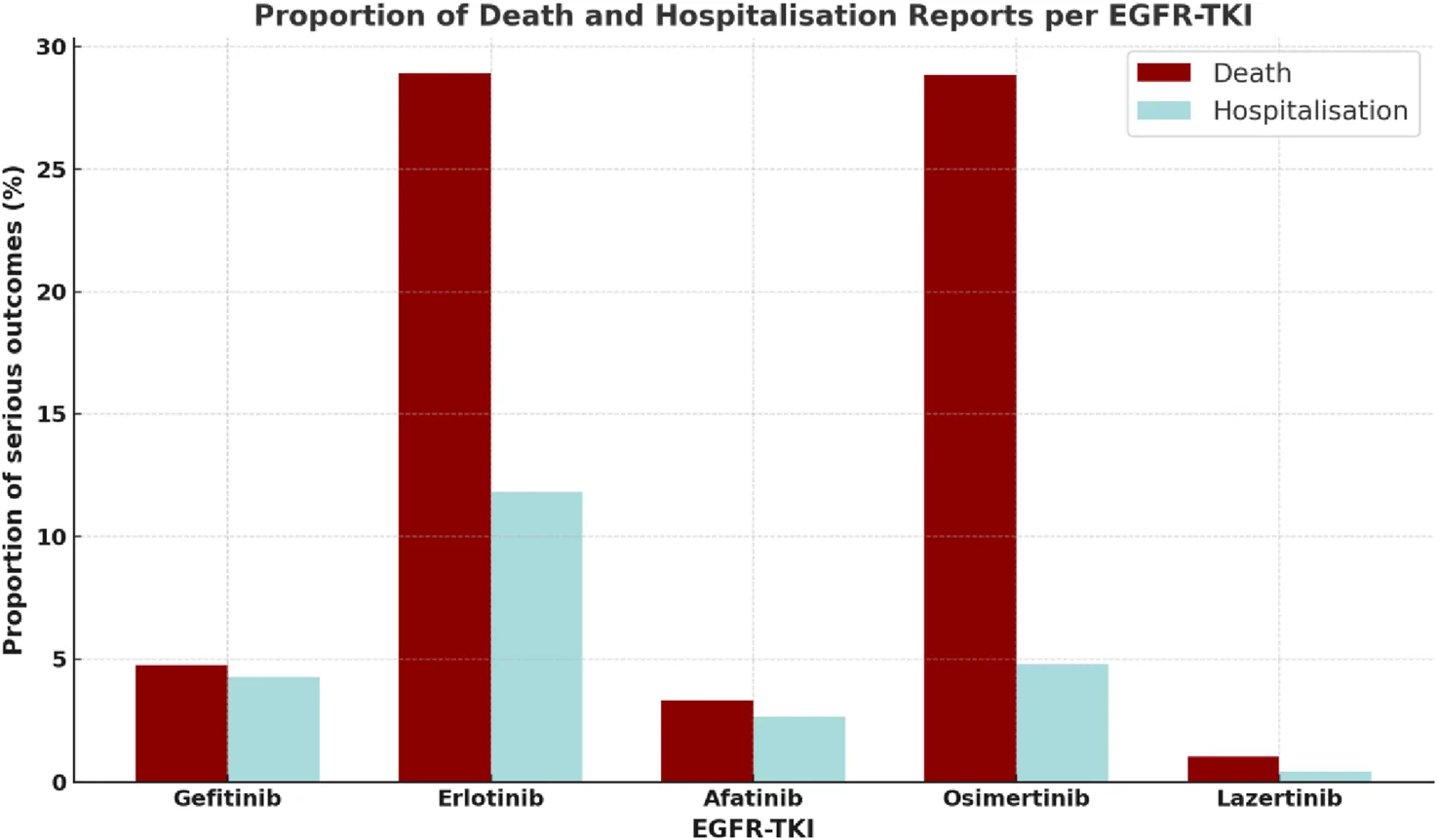
Proportion of serious adverse events reported for EGFR tyrosine kinase inhibitors (TKIs) based on VigiAccess data. The figure displays the percentage of reports involving death and hospitalisation for Gefitinib, Erlotinib, Afatinib, Osimertinib, and Lazertinib, relative to the total number of reports submitted for each drug.

The proportion of reports classified as serious, particularly those including fatal outcomes and hospitalisation, varied substantially across the five EGFR-targeted tyrosine kinase inhibitors (Figure 2). The highest proportions of reports with a fatal outcome were observed for erlotinib (28.91%, n = 12,367) and osimertinib (28.78%, n = 8,440), whereas gefitinib (4.77%, n = 491), afatinib (3.30%, n = 412), and lazertinib (1.03%, n = 17) showed markedly lower proportions. A similar pattern was observed for reports classified as serious due to hospitalisation, with the highest proportions again reported for erlotinib (11.80%, n = 5,046), followed by osimertinib (4.79%, n = 1,396), gefitinib (4.26%, n = 439), afatinib (2.64%, n = 330), and lazertinib (0.42%, n = 7).

These differences likely reflect variation in treatment setting, disease stage, reporting structure, and regional pharmacovigilance practices rather than treatment-attributable toxicity. Importantly, in spontaneous pharmacovigilance databases such as VigiAccess, outcomes including death and hospitalisation are recorded at the report level and cannot be interpreted as confirmed drug-related adverse reactions. Therefore, these observations should be interpreted cautiously and primarily as indicators of reporting context rather than measures of comparative toxicity severity between agents.

### Distribution and frequency of skin and subcutaneous tissue-related adverse drug reactions among EGFR-TKIs.

The comparative analysis of dermatologic adverse drug reactions (ADRs) reported for five epidermal growth factor receptor tyrosine kinase inhibitors (EGFR-TKIs)—afatinib, gefitinib, erlotinib, osimertinib, and lazertinib—revealed substantial heterogeneity in the reporting patterns of cutaneous ADRs (Table 3). Across all agents, rash and pruritus constituted the most frequently reported skin-related events, although their proportional distribution varied notably by drug.

**TABLE 3:** Most frequently reported cutaneous adverse drug reactions (ADRs) associated with five EGFR tyrosine kinase inhibitors (EGFR-TKIs) based on WHO VigiAccess data. Frequencies are expressed as the percentage of total skin-related ADR reports for each agent.

Gefitinib (n = 2,899)		Erlotinib (n = 13,177)		Afatinib (n = 5,367)		Osimertinib (n = 3,392)		Lazertinib (n = 548)	
ADR	Rate (%)	ADR	Rate (%)	ADR	Rate (%)	ADR	Rate (%)	ADR	Rate (%)
Rash	40.15	Rash	60.19	Rash	56.68	Rash	30.22	Rash	54.01
Pruritus	19.32	Pruritus	10.27	Pruritus	16.19	Pruritus	14.45	Pruritus	24.09
Acne	9.35	Dry skin	9.33	Acne	9.69	Dry skin	13.53	Dermatitis acneiform	6.39
Dermatitis acneiform	8.55	Acne	8.47	Dermatitis acneiform	7.12	Alopecia	7.52	Alopecia	5.11
Dry skin	6.45	Alopecia	6.31	Dry skin	6.97	Onychoclasis	6.63	Urticaria	5.11
Skin exfoliation	4.38	Dermatitis acneiform	6.0	Skin exfoliation	4.73	Nail disorder	5.42	Dermatitis	3.47
Alopecia	3.35	Erythema	3.88	Alopecia	4.42	Erythema	4.16	Dry skin	3.28
Skin fissures	3.31	Skin exfoliation	3.39	Skin fissures	4.27	Acne	3.92	Rash maculo-papular	2.74
Nail disorder	2.38	Skin fissures	1.83	Nail disorder	3.39	Urticaria	3.6	Drug eruption	1.46
Skin disorder	2.1	Skin toxicity	1.68	Skin disorder	2.89	Skin fissures	3.3	Acne	1.09
Skin toxicity	1.93	Blister	1.61	Skin toxicity	2.89	Skin exfoliation	2.83	Palmar-plantar erythrodysaesthesia syndrome	1.09
Dermatitis	1.93	Rash pruritic	1.6	Dermatitis	2.5	Skin disorder	2.65	Rash pruritic	0.91
Erythema	1.9	Skin ulcer	1.54	Erythema	1.96	Dermatitis acneiform	2.54	Skin toxicity	0.91
Urticaria	1.52	Skin disorder	1.42	Urticaria	1.53	Skin toxicity	2.3	Skin ulcer	0.73
Rash maculo-papular	1.52	Urticaria	1.26	Rash maculo-papular	1.42	Skin discolouration	1.92	Blister	0.55
Onychoclasis	1.21	Dermatitis	1.22	Onychoclasis	1.21	Erythema multiforme	1.89	Dermatitis allergic	0.55
Palmar-plantar erythrodysaesthesia syndrome	1.17	Onychoclasis	1.21	Palmar-plantar erythrodysaesthesia syndrome	0.95	Rash pruritic	1.65	Hyperhidrosis	0.55
Skin reaction	1.14	Hair growth abnormal	1.16	Skin reaction	0.88	Palmar-plantar erythrodysaesthesia syndrome	1.56	Cold sweat	0.36
Rash pruritic	1.07	Rash erythematous	1.15	Rash pruritic	0.8	Drug eruption	1.36	Decubitus ulcer	0.36
Blister	1.07	Nail disorder	1.12	Blister	0.73	Blister	1.33	Hyperkeratosis	0.36

Descriptive assessment indicated that erlotinib and afatinib were associated with the highest proportions of reported skin ADRs, particularly acneiform rash, xerosis, and alopecia. In contrast, osimertinib demonstrated a more diversified reporting pattern, with a lower relative proportion of rash but an increased representation of nail and hair disorders. Lazertinib showed a distinct reporting pattern characterized by frequent reports of rash and pruritus, with less frequent reporting of more complex or less common dermatologic events.

Pearson’s χ^2^ tests (Bonferroni-corrected α = 0.0125) were used to compare the proportion of selected cutaneous ADRs between drugs. Rash was more frequently reported with afatinib (56.7%) than with osimertinib (30.2%), χ^2^ (1) = 585.11, p < 0.001, φ = 0.26. Pruritus was less frequently reported with osimertinib (14.5%) than with lazertinib (24.1%), χ^2^ (1) = 32.99, p < 0.001, φ = 0.09. For nail-related events (onychoclasis + nail disorder), osimertinib (5.49%) showed a higher reporting proportion compared with gefitinib (3.59%, χ^2^ (1) = 12.35, p < 0.001, φ = 0.04), erlotinib (3.05%, χ^2^ (1) = 45.93, p < 0.001, φ = 0.05), and lazertinib (2.01%, χ^2^ (1) = 11.28, p < 0.001, φ = 0.05), whereas the difference compared with afatinib (4.60%) did not reach statistical significance (χ^2^ (1) = 3.25, p = 0.071). These findings support the presence of agent-specific reporting patterns for nail-related dermatologic events across EGFR-TKIs.

### Disproportionality analysis based on skin and subcutaneous tissue disorders

A total of 23 preferred terms (PTs) related to cutaneous adverse drug reactions (ADRs) were identified for five EGFR tyrosine kinase inhibitors (EGFR-TKIs): gefitinib (n = 2,899), erlotinib (n = 13,177), afatinib (n = 5,367), osimertinib (n = 3,392), and lazertinib (n = 548) (Table 4). The three most frequently reported cutaneous PTs across all agents were rash, pruritus, and dry skin. Rash represented the dominant dermatologic reporting signal across all agents, with the highest ROR observed for erlotinib (ROR = 7.96), followed by osimertinib (ROR = 5.77), lazertinib (ROR = 5.73), afatinib (ROR = 5.58), and gefitinib (ROR = 3.28), indicating a consistently strong disproportionality signal for this event across EGFR-TKIs.

**TABLE 4:** Comparison of reporting odds ratios (RORs) for the most frequently reported skin-related adverse drug reactions (Preferred Terms, PTs) associated with five EGFR tyrosine kinase inhibitors (gefitinib, erlotinib, afatinib, osimertinib, and lazertinib) based on WHO VigiAccess data.

PT	Gefitinib (n = 2,899)		Erlotinib (n = 13,177)		Afatinib (n = 5,367)		Osimertinib (n = 3,392)		Lazertinib (n = 548)	
	N	ROR (95% CI)	N	ROR (95% CI)	N	ROR (95% CI)	N	ROR (95% CI)	N	ROR (95% CI)
Rash	1,164	0.56 (0.51–0.6)	7,931	1.83 (1.74–1.92)	3,042	1.21 (1.13–1.28)	1,025	0.33 (0.31–0.36)	296	1.04 (0.88–1.23)
Pruritus	560	1.65 (1.5–1.83)	1,353	0.57 (0.53–0.61)	869	1.33 (1.23–1.45)	490	1.11 (1.0–1.23)	132	2.09 (1.71–2.55)
Acne	271	1.2 (1.05–1.38)	1,116	1.12 (1.02–1.23)	520	1.3 (1.17–1.44)	133	0.43 (0.36–0.51)	6	0.12 (0.06–0.28)
Dry skin	248	0.92 (0.8–1.05)	1,230	1.04 (0.96–1.13)	374	0.69 (0.62–0.78)	459	1.68 (1.51–1.88)	18	0.33 (0.21–0.53)
Dermatitis acneiform	187	1.13 (0.96–1.32)	791	1.07 (0.96–1.18)	382	1.32 (1.17–1.49)	86	0.38 (0.31–0.48)	35	1.1 (0.78–1.56)
Alopecia	127	0.72 (0.59–0.86)	831	1.2 (1.08–1.34)	237	0.7 (0.61–0.81)	255	1.38 (1.2–1.59)	28	0.87 (0.59–1.27)
Urticaria	69	1.35 (1.04–1.75)	166	0.5 (0.42–0.61)	82	0.79 (0.62–1.01)	122	2.34 (1.9–2.89)	28	2.99 (2.02–4.43)
Rash pruritic	56	1.39 (1.04–1.85)	211	1.23 (1.0–1.51)	43	0.48 (0.35–0.67)	56	1.16 (0.87–1.54)	5	0.62 (0.25–1.49)
Skin toxicity	44	0.74 (0.54–1.01)	222	0.72 (0.61–0.87)	155	1.68 (1.38–2.03)	78	1.19 (0.93–1.52)	5	0.45 (0.19–1.09)
Blister	31	0.8 (0.55–1.16)	212	1.68 (1.34–2.1)	39	0.5 (0.35–0.69)	45	1.02 (0.75–1.41)	3	0.41 (0.13–1.29)

RORs were calculated using standard disproportionality analysis methods, comparing each PT with all other skin-related ADRs and the total number of non-skin ADRs reported for each drug.

While pruritus was the second most frequently reported ADR for most agents, the strength of the disproportionality signal varied considerably, ranging from ROR = 2.25 for osimertinib to ROR = 0.60 for erlotinib. Conversely, agents such as lazertinib and gefitinib demonstrated intermediate signal strengths (ROR = 1.55 and 1.17, respectively). Dry skin demonstrated a particularly elevated disproportionality signal with osimertinib (ROR = 2.09), while showing weaker signals for the remaining agents (e.g., afatinib: ROR = 0.32; gefitinib: ROR = 0.46).

Lower-frequency ADRs such as blister, urticaria, and skin toxicity revealed modest but detectable disproportionality signals across most agents. For example, urticaria had the highest ROR in osimertinib (0.50), and blister events also showed their highest ROR in osimertinib (0.18), compared with lower values in other drugs. Despite low case counts, palmar–plantar erythrodysaesthesia syndrome demonstrated detectable disproportionality signals in osimertinib (ROR = 0.21) and lazertinib (ROR = 0.05), consistent with previously reported dermatologic safety signals associated with kinase inhibition.

Some PTs, including rash pruritic, nail disorder, and dermatitis acneiform, exhibited low-to-moderate ROR values across most agents, with variation in case burden. For instance, dermatitis acneiform had over 790 cases reported with erlotinib (ROR = 0.34), whereas it occurred less frequently in gefitinib (n = 187; ROR = 0.34) and lazertinib (n = 35; ROR = 0.33).

Overall, erlotinib consistently demonstrated the highest disproportionality signals across multiple dermatologic PTs, suggesting relatively stronger reporting signals for cutaneous ADRs compared with the other agents. In contrast, gefitinib and lazertinib demonstrated comparatively weaker disproportionality signals, although key dermatologic events such as rash and pruritus remained prominent. These findings align with previously reported dermatologic safety signals and support the continued use of pharmacovigilance databases for comparative signal detection.

To further explore the overall dermatologic safety profiles of EGFR-TKIs, a disproportionality analysis was conducted for the full category of skin and subcutaneous tissue disorders using ROR and PRR metrics (Table 5). The analysis demonstrated marked differences in signal strength between agents. Afatinib and erlotinib were associated with the highest disproportionality values, indicating a stronger link with cutaneous adverse drug reactions. Lazertinib and gefitinib showed moderate associations, while osimertinib exhibited a distinctly lower proportional reporting of skin-related events. These findings are consistent with clinical observations and emphasize the relevance of aggregate pharmacovigilance data in informing toxicity expectations across different EGFR-TKIs.

**TABLE 5:** Disproportionality analysis of skin and subcutaneous tissue disorder reports associated with five EGFR-TKIs based on WHO VigiAccess data.

Drug	ROR	ROR 95% CI	PRR	PRR 95% CI
Gefitinib	1.07	1.02–1.12	1.05	1.02–1.08
Erlotinib	1.61	1.57–1.66	1.42	1.39–1.44
Afatinib	2.31	2.22–2.4	1.75	1.71–1.79
Osimertinib	0.26	0.25–0.27	0.35	0.33–0.36
Lazertinib	1.35	1.22–1.5	1.24	1.15–1.33

ROR and PRR values reflect the overall reporting frequency of skin-related adverse events relative to non-skin ADRs for each agent. 95% confidence intervals (CI) are presented for both measures.

### Comparative analysis of adverse events: WHO VigiAccess vs. EMA EudraVigilance databases

To provide a broader perspective on dermatologic adverse-event reporting patterns associated with EGFR tyrosine kinase inhibitors (EGFR-TKIs), we compared adverse-event (AE) data from two major pharmacovigilance systems: WHO’s VigiAccess and the EMA’s EudraVigilance. While the total number of AE reports was consistently higher in WHO—reflecting its broader global coverage—both databases revealed similar qualitative reporting trends. Erlotinib and afatinib showed higher proportions of skin-related AE reports compared with osimertinib, consistent with previously reported dermatologic safety signals. To further contextualize these findings, we examined the ten most frequently reported System Organ Class (SOC) categories across both datasets. Skin, gastrointestinal, and general disorders predominated, with broadly comparable SOC distributions despite regional reporting differences.

**FIGURE 3:**
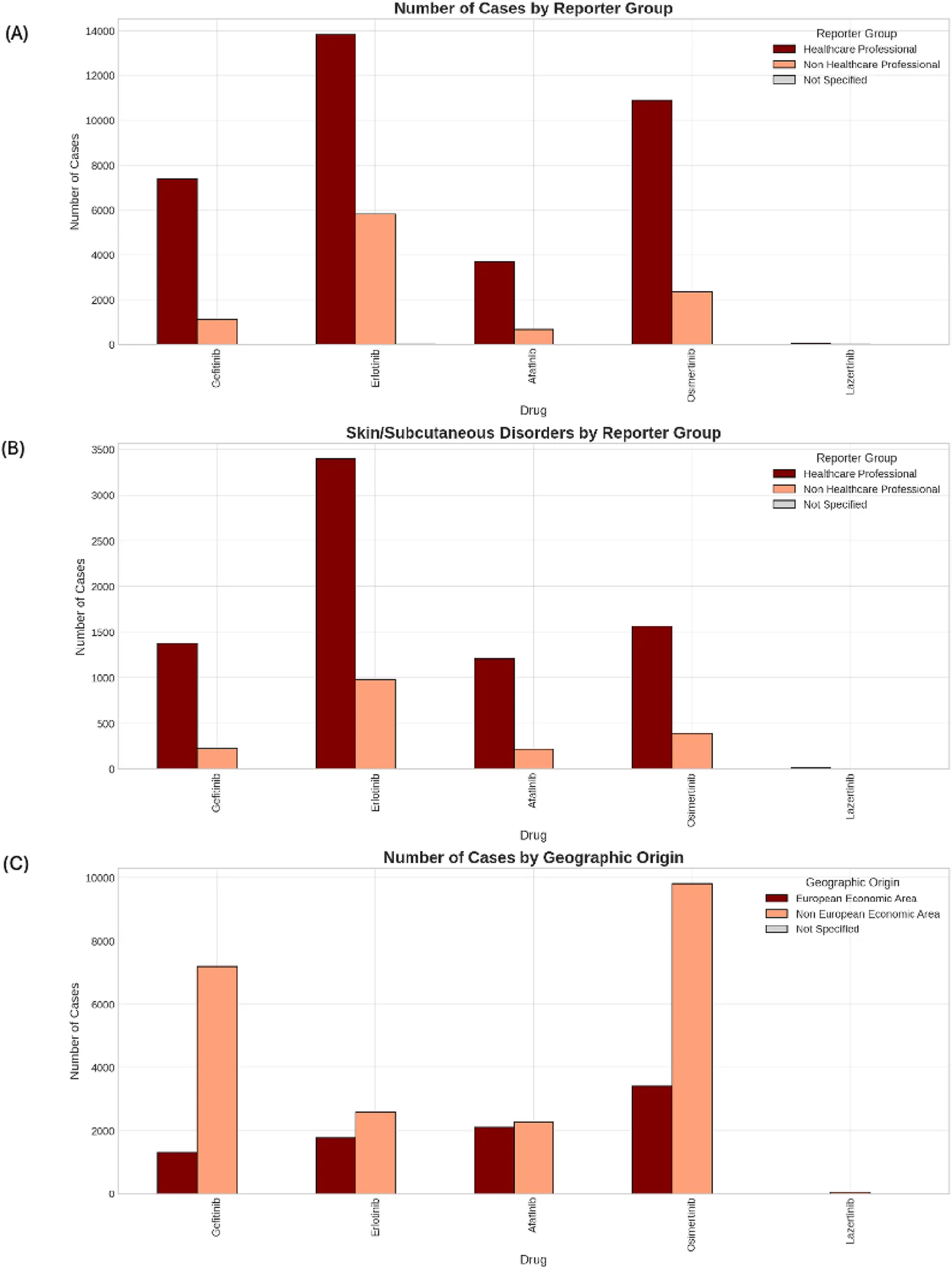
Summary of adverse event reports for EGFR tyrosine kinase inhibitors (TKIs): Gefitinib, Erlotinib, Afatinib, Osimertinib, and Lazertinib. **(A)** The total number of adverse event reports was stratified by the reporter group (healthcare professionals, non-healthcare professionals, and not specified). **(B)** Number of reports related to skin and subcutaneous tissue disorders by reporter group. **(C)** Geographic distribution of all reported adverse events, categorized by origin (European Economic Area, Non-European Economic Area, and not specified). Dark red bars represent reports from healthcare professionals, light salmon bars from non-healthcare professionals, and light grey bars for unspecified reporters or geographic origin. Data retrieved from EudraVigilance as of 19 May 2025.

To assess dermatologic AE reporting patterns across agents, we analyzed data reported to the EMA EudraVigilance database (Figure 3). For each agent, we calculated the proportion of reports related to skin and subcutaneous tissue disorders relative to the total number of adverse-event reports.

The total number of AE reports (n) per drug was as follows: gefitinib (n = 8,507), erlotinib (n = 19,668), afatinib (n = 4,379), osimertinib (n = 13,236), and lazertinib (n = 59). Corresponding numbers of skin-related AE reports were: gefitinib (n = 1,604), erlotinib (n = 4,378), afatinib (n = 1,424), osimertinib (n = 1,961), and lazertinib (n = 15), representing relative reporting proportions of 18.9%, 22.3%, 32.5%, 14.8%, and 25.4%, respectively.

To test whether the proportion of dermatologic AE reports differed significantly across drugs, a chi-square test of independence was conducted. The test revealed a statistically significant association between drug type and the proportion of skin-related AE reports, χ^2^ (4) = 703.82, p < 0.001, indicating that dermatologic adverse-event reporting differed significantly across EGFR-TKIs.

To further explore these differences, we performed pairwise *post hoc* comparisons using z-tests for proportions with Bonferroni correction (adjusted α = 0.005). Significant differences in skin-related AE reporting proportions were observed between multiple drug pairs, including gefitinib vs. erlotinib (p < 0.001), gefitinib vs. afatinib (p < 0.001), erlotinib vs. afatinib (p < 0.001), and afatinib vs. osimertinib (p < 0.001). No significant difference was observed between gefitinib and lazertinib (p = 0.199), likely reflecting the limited number of reports available for the latter.

To further quantify relative differences in dermatologic AE reporting proportions, we calculated reporting odds ratios (OR) and relative reporting ratios (RR) using osimertinib as the reference agent due to its lower proportional representation of skin-related AE reports. Compared with osimertinib, dermatologic AE reporting proportions were higher for afatinib (RR = 2.19; OR = 2.77), erlotinib (RR = 1.50; OR = 1.65), gefitinib (RR = 1.27; OR = 1.34), and lazertinib (RR = 1.72; OR = 1.96).

These findings highlight meaningful differences in dermatologic adverse-event reporting patterns among EGFR-TKIs and indicate relatively higher proportional representation of skin-related adverse-event reports for second-generation inhibitors, particularly afatinib, compared with other agents. Overall, these observations are consistent with previously reported dermatologic safety signals associated with EGFR inhibition.

**FIGURE 4:**
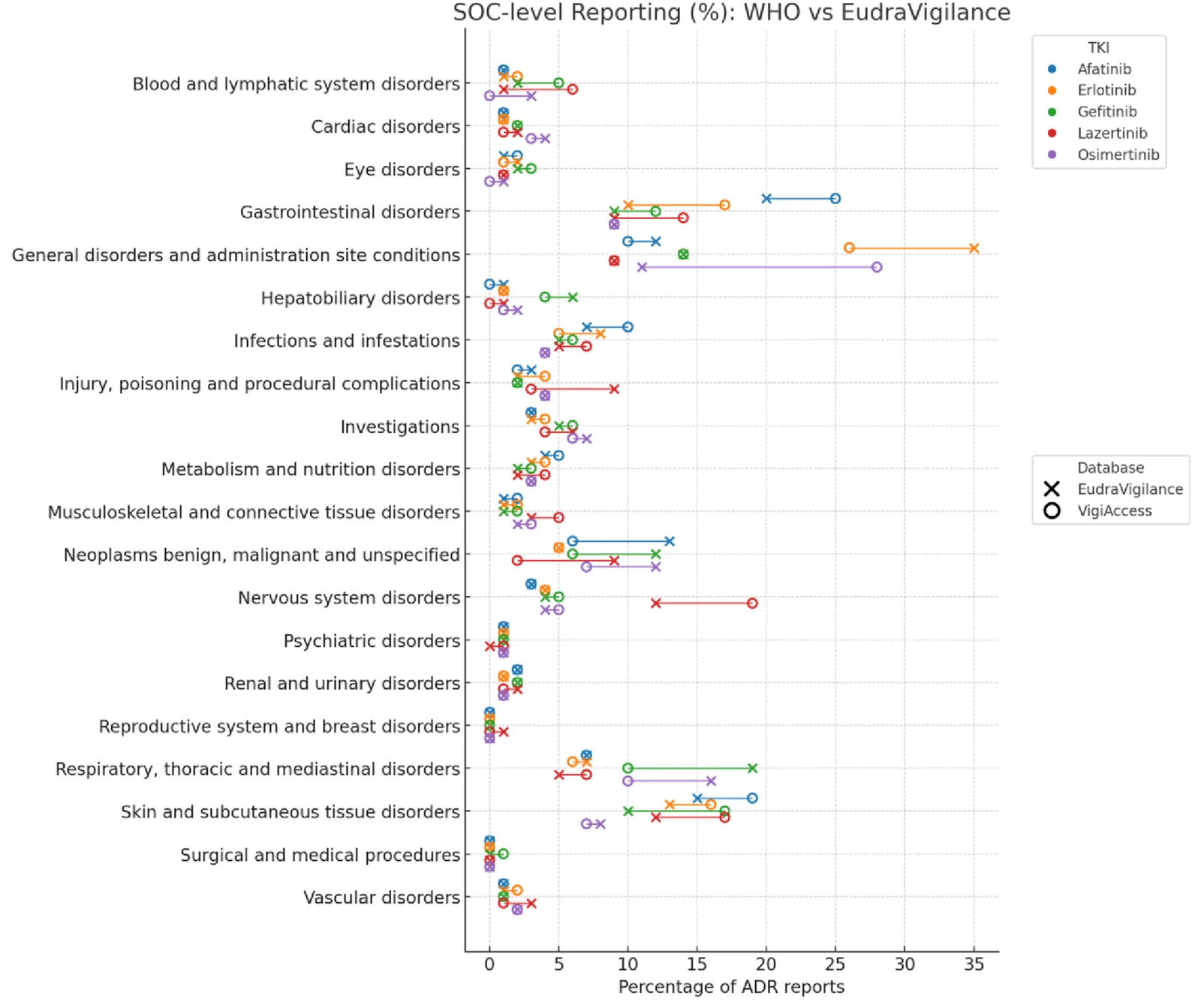
Concordance-gap plot comparing EMA EudraVigilance and WHO VigiAccess reporting across 20 System-Organ-Class (SOC) categories for selected EGFR-targeted tyrosine-kinase inhibitors (TKIs).

A visual comparison via heatmap demonstrated a high degree of overlap between databases in the dominant SOC categories (Figure 4). However, the relative reporting proportions differed: skin-related adverse-event reports accounted for a greater proportion of reports in WHO (28.2%) compared with EMA (21.1%), whereas gastrointestinal events were more frequently represented in EMA (18.4%) than in WHO (14.6%). Other SOC categories, including infections, respiratory disorders, and hepatobiliary disorders, showed broadly comparable reporting distributions across the two databases.

To assess the concordance of adverse-event reporting distributions between the EudraVigilance and WHO VigiAccess databases for five EGFR tyrosine kinase inhibitors (TKIs), we performed a comparative analysis based on the proportional distribution of adverse-event reports across System Organ Class (SOC) categories.

### Dumbbell comparison

A figure was generated to visually compare the percentage distribution of SOC-level adverse event reports across both databases for Gefitinib, Erlotinib, Afatinib, Osimertinib, and Lazertinib (Figure 4).

For each SOC the (x) shows the proportion of adverse-event reports in EudraVigilance, the (○) shows the corresponding proportion in VigiAccess, and the connecting segment visualises the difference between the two databases.

A Wilcoxon signed-rank test was conducted to statistically compare the distribution of adverse-event reports across System Organ Class (SOC) categories between the EudraVigilance and WHO VigiAccess databases for each of the five EGFR tyrosine kinase inhibitors (TKIs): gefitinib, erlotinib, afatinib, osimertinib, and lazertinib. The test was applied to paired percentages derived from both sources, allowing for non-parametric assessment of similarity in reporting distributions. No statistically significant differences were observed for any of the drugs, with p-values well above the conventional threshold of significance (e.g., p = 0.587 for gefitinib, p = 0.200 for erlotinib, and p = 0.935 for lazertinib). These findings indicate a general consistency between the two databases in terms of the overall proportional distribution of adverse-event reports by organ system.

To identify potential outliers in reporting practices between the sources, we additionally calculated the absolute percentage difference (Δ) for each SOC category and determined the most divergent SOC per drug. The most pronounced discrepancy was observed for osimertinib in the category *General disorders and administration site conditions*, with WHO reporting 28% of reports versus 11% in EudraVigilance (Δ = −17 percentage points). A similar category showed divergence for erlotinib (35% vs. 26%; Δ = +9). For gefitinib, the most notable difference was observed in *Respiratory, thoracic and mediastinal disorders* (19% vs. 10%), whereas afatinib and lazertinib both exhibited higher reporting proportions for *Neoplasms benign, malignant and unspecified* in EudraVigilance compared with WHO (13% vs. 6% and 9% vs. 2%, respectively). These findings suggest that while the overall reporting distributions are broadly comparable, individual SOC categories may be subject to variation depending on the reporting system, particularly in categories involving systemic symptoms and oncologic outcomes.

## Discussion

Cutaneous adverse reactions continue to represent a clinical concern in patients treated with EGFR-TKIs. In our analysis, skin toxicities—most frequently acneiform eruptions—were the most commonly reported adverse events across all five agents studied (Boşnak et al., 2018). This pattern is in line with existing clinical data, which consistently show that inhibition of epidermal EGFR signaling disrupts skin homeostasis and leads to characteristic adverse effects (Lee et al., 2024). A meta-analysis reported rash occurrence in 66.5% overall, with afatinib causing rash in 84.8% of patients compared to 62.0% for erlotinib. High-grade adverse events for afatinib were also significant (Recuero et al., 2023). Clinical trial data showed rash incidence for afatinib ranging from 73% to 89% for any grade, with grade 3 rash rates varying per study but reaching up to 16% specifically in the LUX-Lung studies (Ding et al., 2017). In contrast, gefitinib is generally linked to lower rates of rash and fewer severe cases. The reason for these differences is related to gefitinib’s lower affinity for wild-type EGFR in the skin, reducing cutaneous toxicity compared to irreversible blockers such as afatinib (Recuero et al., 2023). The observed differences in the distribution of dermatologic adverse-event reports across sex and age groups should be interpreted cautiously. EGFR-mutant NSCLC is known to occur more frequently in women and in patients with adenocarcinoma histology and never-smoking status compared with EGFR–wild-type disease, and these epidemiologic characteristics likely influence the demographic structure of adverse-event reporting observed in pharmacovigilance datasets (Zhang et al., 2016). Although the association between age and EGFR mutation status varies across populations, the median age at diagnosis typically remains within the sixth to seventh decade of life, similar to NSCLC overall. Therefore, the higher proportion of dermatologic adverse-event reports observed in specific demographic subgroups in our analysis most likely reflects the underlying epidemiology of EGFR-mutant lung cancer rather than true drug-specific susceptibility to cutaneous toxicity. Because spontaneous reporting databases do not provide exposure-adjusted incidence estimates, these findings should be interpreted as descriptive signals consistent with known population characteristics rather than evidence of differential toxicity risk between demographic groups.

The cited clinical trial data are consistent with our pharmacovigilance findings, in which rash accounted for 60.2% of skin-related reports for erlotinib, 56.7% for afatinib, and 40.1% for gefitinib. These results confirm the higher risk of dermatologic toxicity associated with first- and second-generation EGFR-TKIs compared with gefitinib. Third-generation EGFR inhibitors, such as osimertinib, were developed to overcome resistance mutations such as T790M and to improve safety profiles compared with earlier-generation TKIs (Płużański and Piórek, 2016). A key advantage of osimertinib is its selectivity for mutant EGFR forms over wild-type EGFR, which is widely expressed in normal tissues such as the skin. In the FLAURA trial, rash occurred in 58% of patients treated with osimertinib compared with 78% in those receiving first-generation agents (McCoach and Jimeno, 2016). Grade ≥3 rash was uncommon, reported in approximately 1% of cases. These data support the concept that increased selectivity may mitigate, although not completely prevent, dermatologic side effects. This trend was also reflected in our pharmacovigilance analysis, where rash accounted for 30.2% of skin-related adverse event reports for osimertinib—substantially lower than for afatinib (56.7%) and erlotinib (60.2%)—further supporting the favorable dermatologic safety profile of third-generation EGFR-TKIs.

To validate the consistency of observed dermatologic safety profiles, we complemented our primary analysis with comparative data from the EMA’s EudraVigilance database. Despite differences in scope, reporting standards, and regional exposure, the results obtained from both pharmacovigilance systems showed notable alignment in the proportional distribution of cutaneous adverse events. This concordance strengthens the external validity of our findings and underscores the reliability of spontaneous reporting data in capturing real-world drug safety patterns. Minor differences observed across System Organ Classes (SOCs) likely reflect variation in drug availability, clinical practice, and national reporting behaviors rather than true pharmacologic discrepancies.

Lazertinib, a third-generation EGFR TKI, generally demonstrates a favorable toxicity profile broadly similar to other third-generation inhibitors. In the LASER301 randomized trial, rash occurred in 46% of patients receiving lazertinib, which was comparable to 50.6% in those treated with gefitinib. However, pruritus was more common with lazertinib (43.7% vs. 35.3%) (Soria et al., 2018). Although the frequency of rash with lazertinib was modestly lower compared with first-generation EGFR inhibitors, the persistence of skin reactions indicates that dermatologic toxicity remains a class effect. Additionally, paresthesias and related sensory symptoms were reported in approximately 33%–39% of patients treated with lazertinib, occurring more frequently than with earlier-generation EGFR TKIs (Soria et al., 2018). These findings highlight the continued need for real-world data to define the safety profiles of newer agents. Post-marketing pharmacovigilance plays an essential role in detecting rare or delayed toxicities not captured during clinical trials (Hwang et al., 2023).

**FIGURE 5:**
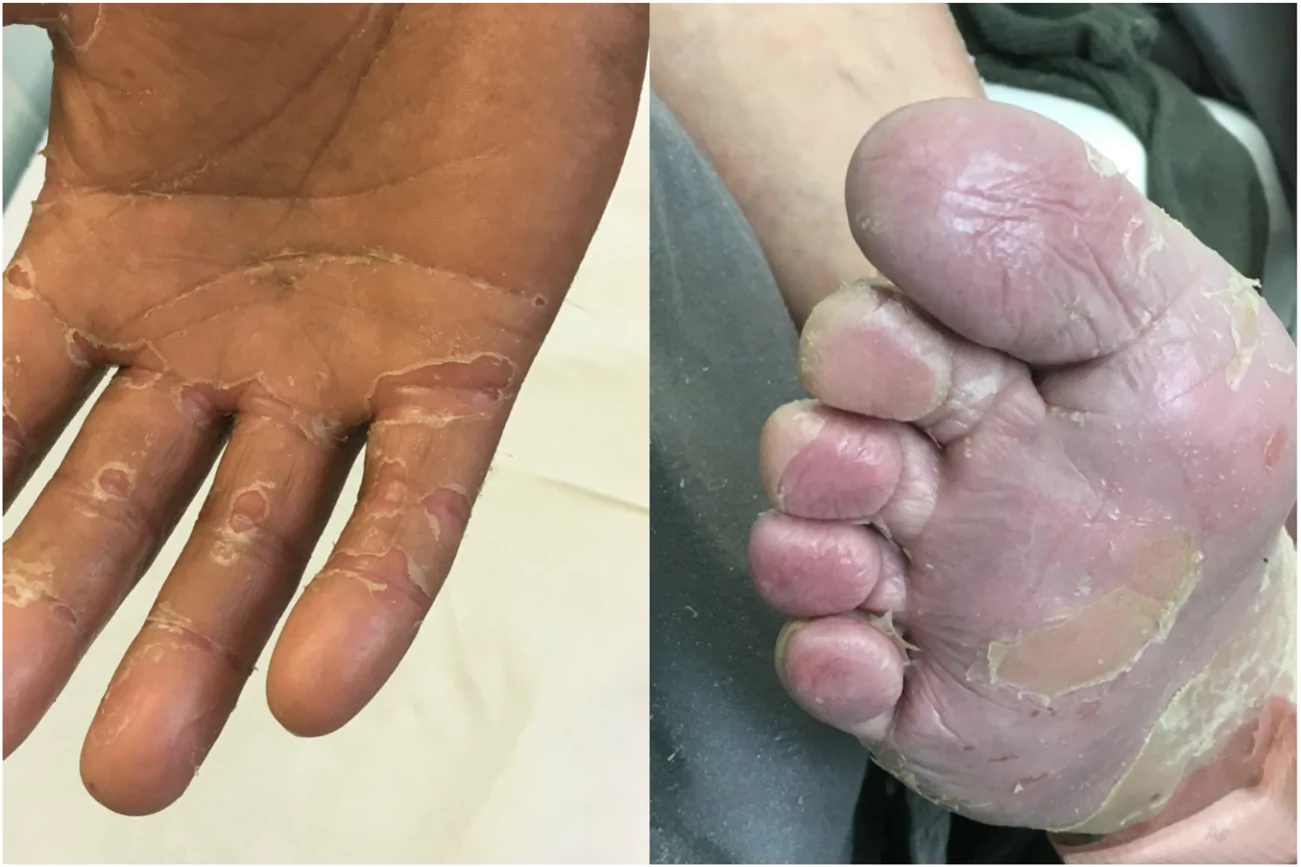
Clinical presentation of palmar-plantar erythrodysaesthesia syndrome (PPES).

While our dataset did not show a clear signal for severe reactions such as Stevens–Johnson syndrome (SJS) or toxic epidermal necrolysis (TEN), both have been documented in patients receiving EGFR-TKIs such as osimertinib and afatinib (Baldo et al., 2018). Their presence in the literature underscores the importance of clinical vigilance and prompt reporting, even in the case of rare events. Among the less frequently reported but clinically relevant cutaneous toxicities is palmar–plantar erythrodysaesthesia syndrome (PPES) (Coleman et al., 2021). Characterized by painful erythema, desquamation, and increased sensitivity of the palms and soles, PPES may impair mobility and reduce quality of life (see Figure 5). Importantly, acral toxicities such as PPES are often underrecognized. This underreporting can occur because patients may not spontaneously report symptoms, or clinicians might overlook examining the feet if patients do not remove footwear during oncology visits. Consequently, thorough and regular skin assessments, including careful inspection of palms and especially soles, are essential in patients receiving EGFR-TKIs to detect and manage these symptoms early and prevent complications.

PPES associated with EGFR-TKIs may develop through mechanisms involving damage to skin cells in acral regions, possibly related to drug concentration in sweat glands and the high turnover rate of keratinocytes in these areas. Management often involves dose adjustments, symptomatic treatments such as moisturizers, avoidance of mechanical stress or heat on affected areas, and patient education to report symptoms promptly. These approaches can help maintain patient quality of life and therapy adherence and further underscore the importance of thorough skin assessments in patients receiving EGFR-TKIs, including acral surfaces, even in the absence of spontaneous complaints.

Skin toxicities, a hallmark adverse event of EGFR-TKI therapy, have significant clinical and psychosocial impacts that extend far beyond their frequency. Even mild dermatologic symptoms—such as pruritus, xerosis, and visible rash—can cause persistent discomfort and pain, lower self-esteem, contribute to body image concerns, disrupt daily activities, sleep, and social interactions, and impair quality of life, particularly in patients with chronic or visible skin reactions (Lee et al., 2024; Zheng et al., 2012). Surveys of oncologists indicate that up to 76% have interrupted or withheld EGFR-TKI therapy because of skin rash at some point during treatment, and nearly one-third report permanent discontinuation in response to severe dermatologic toxicities (Li et al., 2022; Tseng et al., 2020). These consequences may reduce adherence to therapy and overall satisfaction with cancer care (Lacouture et al., 2013). Thus, patient education about the likelihood and management of skin reactions should be standard practice prior to EGFR-TKI initiation (Tseng et al., 2020). Dermatologic side effects often develop early, typically within the first 2 weeks of therapy (Ding et al., 2017), highlighting the need for timely intervention. Accordingly, current clinical guidelines recommend a proactive strategy that includes patient education, routine use of emollients, photoprotection, and prophylactic antibiotics such as doxycycline or minocycline (Melosky et al., 2015). Incorporating multidisciplinary support, including dermatologists and oncology pharmacists, can further improve symptom control and reduce the likelihood of treatment disruption (Lacouture et al., 2011; Napp et al., 2019).

The broader context of these findings emphasizes the evolving importance of pharmacovigilance in oncology. As newer agents such as lazertinib enter routine clinical use, ongoing safety monitoring becomes essential. Bridging the gap between controlled clinical trials and everyday practice requires active participation from the clinical team and robust reporting systems. Physicians and pharmacists play complementary roles in this process: clinicians identify and manage adverse events, while pharmacists engage in patient education, monitor for early signs of toxicity, and ensure proper documentation and submission to safety databases. Underreporting remains a challenge but can be mitigated by team-based pharmacovigilance approaches. Routine questioning, structured toxicity assessments, and use of global reporting platforms such as VigiBase contribute to early detection and improve the quality of care.

Based on our comparative analysis, skin toxicity remains a consistent feature of EGFR-TKI therapy. Differences in severity and type between agents should inform monitoring strategies. Clinicians are encouraged to implement preemptive skin care protocols at the start of treatment and maintain a low threshold for intervention. With early recognition and coordinated management, patients can often remain on therapy and achieve the intended oncologic benefits.

From a clinical perspective, the observed differences in dermatologic adverse-event reporting patterns across EGFR-TKIs are consistent with known mechanistic differences between agents and may help inform individualized supportive care strategies. First- and second-generation EGFR-TKIs such as erlotinib and afatinib inhibit both mutant and wild-type EGFR, which is strongly expressed in keratinocytes and sebaceous glands and plays a central role in epidermal homeostasis. As a result, broader EGFR inhibition is associated with a higher proportional burden of dermatologic adverse-event reports. In contrast, third-generation agents such as osimertinib and lazertinib demonstrate greater selectivity for mutant EGFR and reduced activity against wild-type receptors, which likely contributes to their comparatively lower proportional representation of cutaneous adverse-event reports.

These differences have practical implications for clinical management. Patients treated with first- and second-generation EGFR-TKIs may benefit particularly from early dermatologic monitoring, prophylactic skin-care strategies, and patient education regarding expected cutaneous reactions. Conversely, although third-generation agents generally show more favorable dermatologic reporting patterns, nail and hair disorders remain clinically relevant and should be actively monitored during long-term therapy. Integration of oncology pharmacists and dermatologists into routine care pathways may further improve early detection of cutaneous toxicities, support treatment adherence, and reduce the risk of dose modification or treatment interruption.

Taken together, these findings support the use of pharmacovigilance-based safety signal analyses as a complementary source of real-world evidence that can inform individualized toxicity monitoring strategies and multidisciplinary supportive care in patients receiving EGFR-targeted therapy.

## Conclusion

This study provides a comprehensive real-world assessment of dermatologic adverse-event reporting associated with EGFR tyrosine kinase inhibitors using two major pharmacovigilance databases. Skin toxicities, particularly acneiform rash and pruritus, emerged as the most frequently reported events across all agents. The observed concordance between WHO VigiAccess and EMA EudraVigilance reporting systems supports the reproducibility and external validity of these dermatologic safety signals. Although most reported cutaneous reactions were consistent with known class-related effects of EGFR inhibition, less frequently reported but clinically relevant toxicities such as palmar–plantar erythrodysaesthesia warrant increased clinical awareness.

These findings highlight the importance of systematic skin monitoring and multidisciplinary management strategies to support treatment adherence and optimize patient outcomes during EGFR-TKI therapy. Importantly, the purpose of this study was not to rank EGFR-TKIs according to toxicity severity but to identify reproducible dermatologic safety signal patterns across pharmacovigilance systems that may support individualized toxicity monitoring in routine clinical practice. Earlier-generation inhibitors demonstrated consistently higher proportional representation of dermatologic adverse-event reports, whereas third-generation inhibitors showed more diversified cutaneous reporting patterns with relatively greater involvement of nail- and hair-related events. These observations may assist clinicians in anticipating the spectrum of dermatologic toxicities most likely to occur during treatment and in implementing proactive supportive-care strategies tailored to expected toxicity patterns rather than relying solely on trial-based incidence estimates.

## Data Availability

The raw data supporting the conclusions of this article will be made available by the authors, without undue reservation.
