# Supplementary material for: Global pharmacovigilance analysis of dermatologic toxicity in EGFR-TKI–treated NSCLC: a cross-database real-world study
Source: Front Pharmacol. 2026 Aug 12;17:1789598. doi: 10.3389/fphar.2026.1789598 (PMC13506688; doi:10.3389/fphar.2026.1789598)
Supplement: Supplementary file 1 [file Supplementaryfile1.docx]

**Supplementary table 1.** Adverse Drug Reaction (ADR) Reports by System Organ Class (SOC) for Five EGFR Tyrosine Kinase Inhibitors in the WHO VigiAccess Database (as of May 2025)

| **System organ class (SOC)** | **Gefitinib** | **Erlotinib** | **Afatinib** | **Osimertinib** | **Lazertinib (N=1650)** |
| --- | --- | --- | --- | --- | --- |
| **Blood and lymphatic system disorders** | 413 (2%) | 1367 (2%) | 259 (1%) | 1248 (3%) | 44 (1%) |
| **Cardiac disorders** | 263 (2%) | 1056 (1%) | 208 (1%) | 1364 (3%) | 18 (1%) |
| **Eye disorders** | 275 (2%) | 1688 (2%) | 417 (1%) | 643 (1%) | 31 (1%) |
| **Gastrointestinal disorders** | 2161 (12%) | 10025 (12%) | 7027 (25%) | 4073 (9%) | 449 (14%) |
| **General disorders and administration site conditions** | 2510 (14%) | 20773 (26%) | 2782 (10%) | 13018 (28%) | 276 (9%) |
| **Hepatobiliary disorders** | 685 (4%) | 688 (1%) | 134 (0%) | 494 (1%) | 15 (0%) |
| **Infections and infestations** | 994 (6%) | 4243 (5%) | 2740 (10%) | 2098 (4%) | 230 (7%) |
| **Injury, poisoning and procedural complications** | 356 (2%) | 3103 (4%) | 585 (2%) | 1732 (4%) | 100 (3%) |
| **Investigations** | 994 (6%) | 3102 (4%) | 844 (3%) | 2582 (6%) | 142 (4%) |
| **Metabolism and nutrition disorders** | 570 (3%) | 2885 (4%) | 1421 (5%) | 1356 (3%) | 122 (4%) |
| **Musculoskeletal and connective tissue disorders** | 388 (2%) | 1686 (2%) | 496 (2%) | 1368 (3%) | 161 (5%) |
| **Neoplasms benign, malignant and unspecified** | 1052 (6%) | 4012 (5%) | 1845 (6%) | 3403 (7%) | 61 (2%) |
| **Nervous system disorders** | 812 (5%) | 3225 (4%) | 969 (3%) | 2217 (5%) | 609 (19%) |
| **Psychiatric disorders** | 247 (1%) | 1149 (1%) | 309 (1%) | 596 (1%) | 37 (1%) |
| **Renal and urinary disorders** | 300 (2%) | 908 (1%) | 450 (2%) | 501 (1%) | 40 (1%) |
| **Reproductive system and breast disorders** | 39 (0%) | 159 (0%) | 83 (0%) | 100 (0%) | 9 (0%) |
| **Respiratory, thoracic and mediastinal disorders** | 1782 (10%) | 4963 (6%) | 1880 (7%) | 4860 (10%) | 215 (7%) |
| **Skin and subcutaneous tissue disorders** | 2899 (17%) | 13777 (16%) | 5367 (19%) | 3392 (7%) | 548 (17%) |
| **Surgical and medical procedures** | 38 (0%) | 365 (0%) | 87 (0%) | 160 (0%) | 3 (0%) |
| **Vascular disorders** | 252 (1%) | 1463 (2%) | 237 (1%) | 834 (2%) | 36 (1%) |

**Supplementary Table 2.** Top 20 most frequently reported specific adverse drug reactions (ADRs) for five EGFR tyrosine kinase inhibitors (Gefitinib, Erlotinib, Afatinib, Osimertinib, and Lazertinib) based on VigiAccess pharmacovigilance data.

| **Gefitinib** | | **Erlotinib** | | **Afatinib** | | **Osimertinib** | | **Lazertinib** | |
| --- | --- | --- | --- | --- | --- | --- | --- | --- | --- |
| **ADR** | **Rate (%)** | **ADR** | **Rate (%)** | **ADR** | **Rate (%)** | **ADR** | **Rate (%)** | **ADR** | **Rate (%)** |
| **Rash** | 11.29% | **Death** | 28.91% | **Diarrhoea** | 45.53% | **Death** | 28.78% | **Rash** | 17.99% |
| **Diarrhoea** | 10.67% | **Rash** | 18.55% | **Rash** | 24.35% | **Diarrhoea** | 6.94% | **Paraesthesia** | 15.26% |
| **Pruritus** | 5.43% | **Diarrhoea** | 12.96% | **Stomatitis** | 15.30% | **Malignant neoplasm progression** | 5.37% | **Diarrhoea** | 9.33% |
| **Interstitial lung disease** | 5.10% | **Nausea** | 4.81% | **Paronychia** | 12.61% | **Interstitial lung disease** | 5.21% | **Pruritus** | 8.00% |
| **Malignant neoplasm progression** | 4.83% | **Fatigue** | 4.55% | **Malignant neoplasm progression** | 9.91% | **Rash** | 3.54% | **Neuropathy peripheral** | 6.72% |
| **Death** | 4.76% | **Disease progression** | 3.42% | **Decreased appetite** | 7.14% | **Fatigue** | 3.06% | **Stomatitis** | 6.48% |
| **Nausea** | 3.62% | **Decreased appetite** | 3.41% | **Pruritus** | 6.95% | **Decreased appetite** | 2.72% | **Hypoaesthesia** | 5.09% |
| **Dyspnoea** | 3.55% | **Pruritus** | 3.17% | **Nausea** | 6.15% | **Dyspnoea** | 2.40% | **Headache** | 4.91% |
| **Vomiting** | 3.10% | **Vomiting** | 3.13% | **Vomiting** | 5.09% | **Nausea** | 2.31% | **Decreased appetite** | 4.78% |
| **Decreased appetite** | 2.98% | **Drug ineffective** | 3.02% | **Acne** | 4.16% | **Pneumonitis** | 2.24% | **Nausea** | 4.36% |
| **Drug resistance** | 2.75% | **Malignant neoplasm progression** | 2.96% | **Death** | 3.29% | **Asthenia** | 2.01% | **Muscle spasms** | 4.30% |
| **Hepatic function abnormal** | 2.73% | **Dyspnoea** | 2.93% | **Mucosal inflammation** | 3.28% | **Drug resistance** | 1.99% | **Constipation** | 4.18% |
| **Acne** | 2.63% | **Dry skin** | 2.88% | **Dehydration** | 3.27% | **Drug ineffective** | 1.84% | **Pneumonia** | 4.06% |
| **Disease progression** | 2.60% | **Off label use** | 2.76% | **Dermatitis acneiform** | 3.05% | **Thrombocytopenia** | 1.81% | **Alanine aminotransferase increased** | 3.63% |
| **Asthenia** | 2.42% | **Asthenia** | 2.65% | **Asthenia** | 3.02% | **Pneumonia** | 1.73% | **Asthenia** | 3.39% |
| **Dry skin** | 2.41% | **Acne** | 2.61% | **Dry skin** | 2.99% | **Pruritus** | 1.67% | **Dizziness** | 3.39% |
| **Pneumonia** | 2.18% | **Pneumonia** | 2.29% | **Fatigue** | 2.85% | **Cough** | 1.62% | **Paronychia** | 3.27% |
| **Fatigue** | 1.98% | **Weight decreased** | 2.08% | **Dyspnoea** | 2.38% | **Dry skin** | 1.58% | **Dyspnoea** | 2.97% |
| **Cough** | 1.93% | **Malaise** | 1.95% | **Weight decreased** | 2.09% | **Vomiting** | 1.52% | **Fatigue** | 2.91% |
| **Pyrexia** | 1.87% | **Alopecia** | 1.94% | **Pneumonia** | 2.05% | **Weight decreased** | 1.48% | **Aspartate aminotransferase increased** | 2.91% |
